# Assessing and Correcting Topographic Effects on Forest Canopy Height Retrieval Using Airborne LiDAR Data

**DOI:** 10.3390/s150612133

**Published:** 2015-05-26

**Authors:** Zhugeng Duan, Dan Zhao, Yuan Zeng, Yujin Zhao, Bingfang Wu, Jianjun Zhu

**Affiliations:** 1 Key Laboratory of Digital Earth Science, Institute of Remote Sensing and Digital Earth (RADI), Chinese Academy of Science, Haidian District, Beijing 100094, China; E-Mails: dzg47336628@163.com (Z.D.); zhaodan@radi.ac.cn (D.Z.); zhaoyj@radi.ac.cn (Y.Z.); wubf@radi.ac.cn (B.W.); 2 School of GeoSciences and Info-Physics, Central South University, Changsha 410083, China; E-Mail: zjj@mail.csu.edu.cn; 3 School of Sciences, Central South University of Forestry and Technology, Changsha 410004, China

**Keywords:** LiDAR, canopy height point cloud, Dinghushan National Nature Reserve, multi-resolution segmentation, topography

## Abstract

Topography affects forest canopy height retrieval based on airborne Light Detection and Ranging (LiDAR) data a lot. This paper proposes a method for correcting deviations caused by topography based on individual tree crown segmentation. The point cloud of an individual tree was extracted according to crown boundaries of isolated individual trees from digital orthophoto maps (DOMs). Normalized canopy height was calculated by subtracting the elevation of centres of gravity from the elevation of point cloud. First, individual tree crown boundaries are obtained by carrying out segmentation on the DOM. Second, point clouds of the individual trees are extracted based on the boundaries. Third, precise DEM is derived from the point cloud which is classified by a multi-scale curvature classification algorithm. Finally, a height weighted correction method is applied to correct the topological effects. The method is applied to LiDAR data acquired in South China, and its effectiveness is tested using 41 field survey plots. The results show that the terrain impacts the canopy height of individual trees in that the downslope side of the tree trunk is elevated and the upslope side is depressed. This further affects the extraction of the location and crown of individual trees. A strong correlation was detected between the slope gradient and the proportions of returns with height differences more than 0.3, 0.5 and 0.8 m in the total returns, with coefficient of determination *R^2^* of 0.83, 0.76, and 0.60 (n = 41), respectively.

## 1. Introduction

Airborne light detection and ranging (LiDAR) has become an effective and reliable way to map terrain and retrieve forest structural parameters [1]. In fact, forest managers have found LiDAR to be of great utility when compared with traditional methods as a way to obtain forest information. Many important forest parameters can be obtained directly or indirectly from LiDAR data, such as tree height, crown width, diameter, canopy density, volume and biomass [2,3,4]. Before forest parameters obtained by LiDAR inversion can be applied, understanding the causes and magnitude of errors of such parameters is essential. A variety of factors can cause errors in LiDAR-based estimates including the terrain, forest structure and point cloud filtering algorithms; variations in topography also play a key role in data extraction.

A series of studies have shown the accuracy of LiDAR-derived digital elevation models (DEMs) and tree parameters generally decreases as slope gradient increases. When the slope gradient increases from 15.6° to 37.6°, the vertical Root Mean Square Error of tree height extraction increases from 0.576 m to 0.901 m [5]. Hodgson and Bresnahan [6] examined the effects of topography on tree height and spatial structure of forest within a small plot. Gatziolis *et al.* [7] studied the accuracy of an airborne LiDAR-derived DEM in a coniferous forest area with high biomass. Their results showed that DEM accuracy was mainly affected by the ground slope and sensor accuracy; increasing slope gradient resulted in reduced tree height because LiDAR inversion and DEM errors led to forest volume errors.

Breidenbach *et al.* [8] investigated tree height retrieved from LiDAR and InSAR data and concluded that slopes generally impacted the estimation of tree height. They also suggested that as the slope gradient increased, models neglecting slopes would overestimate tree height, and noted that this could be corrected by a slope coefficient that would allow a more accurate estimation of tree height.

Complex forest habitats and systems require accurate DEM extraction as the basis for forest parameter inversion. The accuracy of DEM extraction in turn is related to ground point classification and DEM interpolation. Complex terrains tend to cause misclassifications and missed points; therefore, the ground point cloud does not always reflect the actual terrain conditions, resulting in lower DEM accuracy, thus affecting parameter extraction [9]. Evans *et al.* [4] studied the effect of ground point misclassifications on the extraction of vegetation height. Interpolation is typically required to generate a DEM. However, the precision of the interpolation is again dependent on terrain complexity [10]. Much attention has been placed on the impact of DEM interpolation on the extraction of canopy height and tree height. Filtering algorithms used to generate LiDAR-based DEMs for complex forests mainly include Iterative Approximation [11,12,13], Progressive Densification [14], Morphological Filtering [15,16,17], and Multi-scale Curvature Classification (MCC) [18].

Forest canopy height describes the top of the vegetated canopy as well as the vertical and horizontal distribution of the canopy; it is the key to assessing forest parameters [2,19]. An accurate estimate of canopy height significantly affects the inversion of forest parameters. Canopy height can be used to directly measure individual tree parameters such as crown vertices, crown diameter, and height to the first live branch [20]. Additionally, canopy height can also be used to calculate the Diameter at Breast Height (DBH) of individual trees, the average DBH of a forest stand, as well as stand volume and biomass through relative growth equations. In a word, canopy height is the basis for individual tree parameters such as tree height, crown diameter, height to the first live branch, DBH, canopy density, and other parameters including parameters related to forest structure, volume and biomass. Therefore, the accurate extraction of canopy height is the key to accurate estimation of forest structure parameters and biomass retrieval. Two methods used to express canopy height are the discrete normalized point cloud method and grid canopy height models (CHM). The former is obtained directly by subtracting DEM data from filtered and classified point cloud data, while the latter was produced by subtracting DEM data from a Digital Surface Model (DSM); a DSM can be generated by interpolating filtered and classified point cloud data [21,22]. Therefore, a CHM is also known as a Normalized Digital Surface Model (nDSM), Digital Canopy Height Model (DCHM) or Digital Canopy Model (DCM) [23].

Both the normalized point cloud and CHMs are subject to the influence of terrain. Previous researchers have paid a considerable amount of attention to the influence of terrain on DEMs and the impact of classifications and filtering algorithms on forest parameter extraction, but few studies have explored the process of obtaining normalized point cloud data or CHMs. Canopy height differences are generated by subtracting a DEM from a DSM or the raw point cloud data. This paper proposes a method for terrain correction of canopy height based on individual tree crown segmentation. The aim is to assess and correct topographical effects on forest canopy height retrieval using airborne LiDAR data.

## 2. Study Area and Data

### 2.1. Study Area

**Figure 1:**
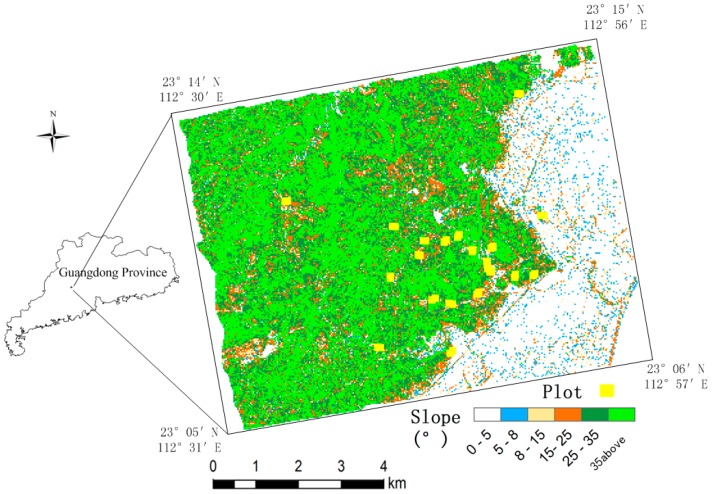
Distribution of slope in study area and plots.

The study area (Figure 1) is located in Dinghushan National Nature Reserve (23°05′, 23°15′N, 112°30′, 112°57′E), Dinghu District of Zhaoqing City, in the west central part of Guangdong Province, China. The area has a mean elevation of 545 m (minimum, 14.1 m; maximum, 1000.3 m). A rather complex terrain characterises the topography of the study area. The mountains in this hilly area run downhill from southwest to northeast. The area is covered by steep slopes, among which 44.7% are between 0–5°; 2.0%, 5–8°; 3.3%, 8–5°; 6.8%, 15–25°; 17.1%, 25–35°; and 26.1% are ≥35°. Vegetation in the area falls into six categories: evergreen broadleaf forest, coniferous and broadleaf mixed forest (here after referred to as “mixed forest”), tropical evergreen coniferous forest, montane evergreen shrub, montane evergreen bushes, and anthropogenic vegetation. The main tree species in the study area were *Pinus massoniana*, *Schima superba*, *Castanopsis chinensis*, and *Eucalyptus robusta*.

### 2.2. LiDAR Data

The LiDAR survey was carried out on 24 December 2012 using LiteMapper 6800 (Ingenieur Geshellshaft für Interfaces mbH (IGI), Kreuztal, Germany, www.igi.eu); the main technical parameters are presented in Table 1. LiteMapper 6800 is capable of providing full waveform LiDAR data and high-resolution aerial images simultaneously because it is equipped with Rollei PRO 6.5 megapixels digital cameras (Rollei, Braunschweig, Germany). A Y-5 aircraft was used as an aerial platform. The aircraft covered a total area of 60 km^2^, with an average speed of 160 km/h, an absolute cruising altitude of 1300 m, an average relative altitude of 750 m, and an average point cloud density of 15.0 p/m^2^.

**Table 1:** Main technical parameters of LiteMapper 6800.

Device Type	LiteMapper 6800
Pulse repetition frequency	Up to 400 KHz
Laser wavelength	1550 ns
Pulse length	3.5 ns
Laser beam divergence	≤0.5 mrad
Multiple target separation within single shot	0.6 m
Return pulse width resolution	0.15 m
Scan pattern	Parallel scan
Scan angle range	±30°
Angle readout resolution	0.001°
Ground sample spot diameter	0.24 m (@800 m)
Horizontal accuracy	0.08 m (@800 m)
Vertical accuracy	0.04 m (@800 m)

### 2.3. Field Inventory Data

**Figure 2:**
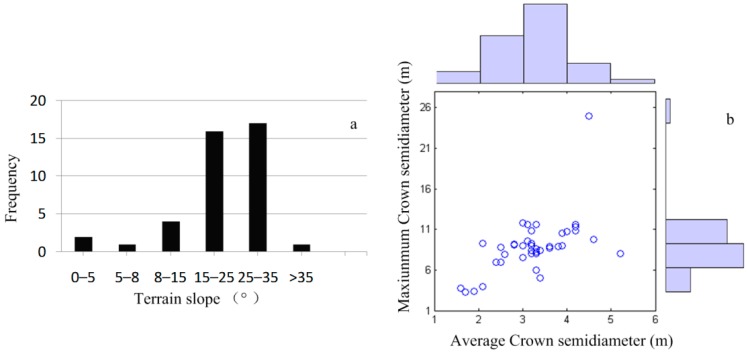
Distribution of terrain slope in the plots, average and maximum crown semidiameter. (**a**) terrain slope; (**b**) average and maximum crown semidiameter.

The ground-truth data were collected from 16 November 2012 to 9 December 2012. A total of 41 plots (30 × 30 m^2^), with most slopes between 8–40° (Figure 2a), were established, including four in coniferous forests, 22 in broadleaf forests, and 15 in mixed forests. Parameters measured mainly included: DBH of individual trees with a DBH >5 cm (DBH is measured from 1.3 m above ground), tree height, height to the first live branch, crown diameter, canopy density, slope gradient and aspect; all served as samples and evidence for LiDAR-based biomass inversion and biodiversity research. Plot area was measured by a forest compass combined with a measuring tape. Slope gradients were also determined by a forest compass. Angular point coordinates of plots were determined by the wide area differential signals of a Trimble3000 handheld GPS (Trimble, Sunnyvale, CA, USA), with sub-meter nominal accuracy. Individual tree DBHs were measured by a DBH tape; tree height and height to the first live branch were measured by a laser altimeter; crown semidiameter was measured in east-west and north-south directions with a measuring tape. Figure 2b shows that the average crown semidiameter in the plots was 1.6–5.2 m, and maximum crown semidiameter was 3.3–25.0 m, respectively.

Plot number 34, a broadleaf forest plot with *S. superba*, *Mallotus paniculatus* and *Ficus variolosa* as the dominant tree species, is cited again as an example. This plot had average elevation 562.4 m and average slope 31.2°. Field surveys detected a total of 98 trees, with an average height of 7.86 m (maximum, 13.5 m; minimum, 3.6 m). The average, maximum, minimum DBH of all trees were 9.2 cm, 37.0 cm and 5.0 cm, respectively, average crown at 4.1 m (maximum, 16.8 m; minimum, 1.5 m). The plot point cloud comprised 14,047 points.

## 3. Methodology

### 3.1. Terrain Impacts on Canopy Height

Both the canopy height point cloud and CHM are by nature elevation differences between the point cloud data, DSM and DEM for the same point in a coordinate plane. For an upright tree, the canopy height should be the difference between the elevation of the canopy and the elevation where the roots enter the ground. However, when the ground surface slopes, which is often the case, or other complex terrain features are present such as ridges, valleys, escarpments, and eroded areas are present, the elevation differences deviate from the actual canopy height.

**Figure 3:**
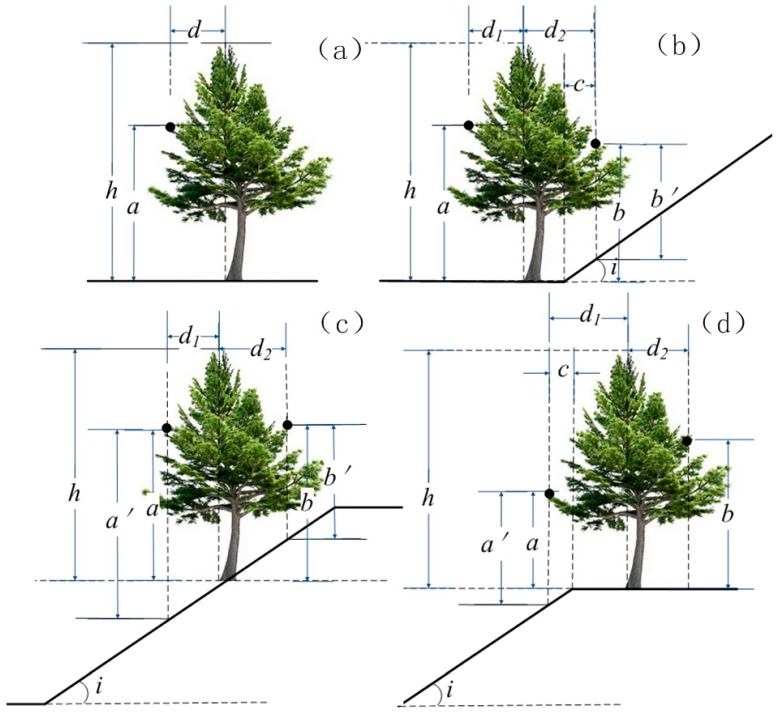
Influence of slope on canopy height.

Figure 3 shows the influence of ground slope on canopy height when the canopy height is calculated as the difference between point cloud data and the DEM. The black dots in the figure represent laser hits on the canopy, where *i* is the angle of the slope gradient. On flat ground, the difference between the point cloud data and DEM reflects the actual canopy height (Figure 3a). The tree is located near the lower end of the slope, with part of its canopy covering the slope (Figure 3b). The difference between the elevation of the laser hit at the left side and DEM is its actual canopy height (*a*). The actual canopy height corresponding to the laser hit at the right side is *b*. However, the difference between the laser hit at the right side and the DEM leads to another height *b’* = *b* – *c* tan(*i*), which represents an error of –*c* tan(*i*).Similarly, when the tree stands on a slope (Figure 3c), canopy height calculated as the differences between DEM and laser hits at the left and the right side are *a’* = *a* + *d_1_* tan(*i*) and *b’* = *b* – *d_2_* tan(*i*), respectively, representing respective differences in *+d_1_* tan(*i*) and –*d_2_* tan(*i*). Additionally, where the tree is located at the top of the slope with its canopy covering part of the slope, the canopy height error at the left side is +*c* tan(*i*) (Figure 3d).

Terrain-induced canopy height differences are mainly determined by the slope gradient and the crown radius in that larger slope gradients and wider crowns lead to greater differences. When the laser hit is on the edge of the crown, and is in the upslope direction, the maximum height difference is expected to be observed. Laser hits farther away from the centre point of the trunk cause larger differences. The maximum differences of canopy height was calculated by the formula *d*/2 tan(*i*) (Table 2), where *d* is the diameter of the crown, and *i* is the slope gradient. Obviously, the influence of terrain on individual tree canopy height measurement is too significant to be ignored.

**Table 2:** Maximum canopy height difference calculated by slope gradient and crown.

	Slope *i* (°)	5	10	20	30	40	50
Crown *d* (m)							
3		0.13	0.26	0.54	0.86	1.26	1.79
5		0.22	0.44	0.91	1.44	2.10	2.97
10		0.44	0.88	1.82	2.88	4.20	5.96
15		0.66	1.32	2.73	4.33	6.29	8.94

**Figure 4:**
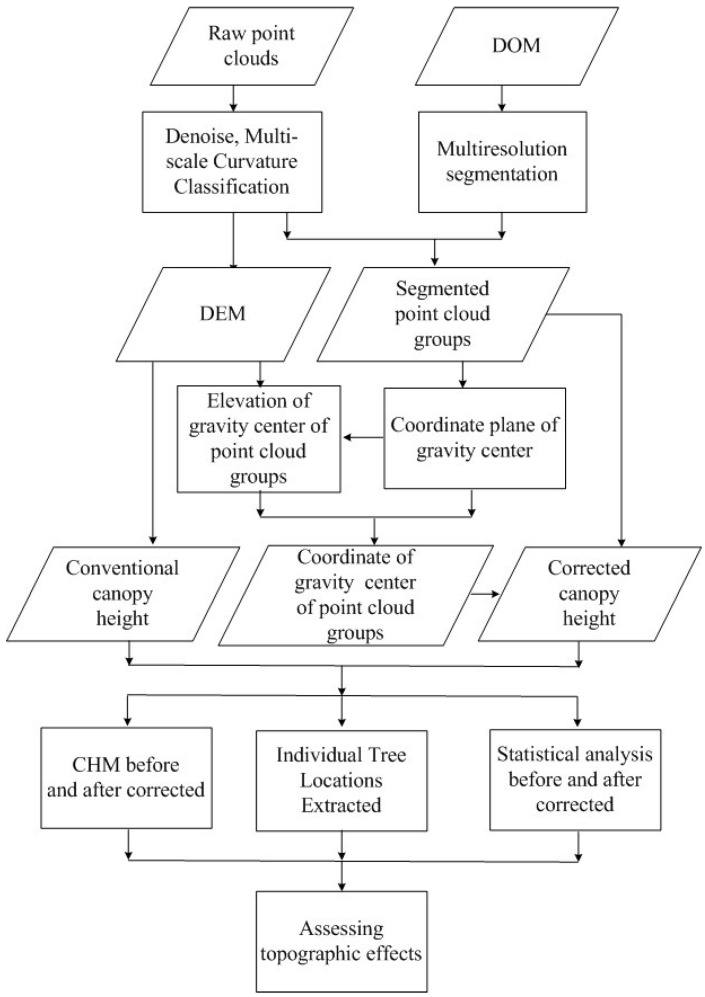
Flowchart of methods used in this study.

This shows that the terrain-induced canopy height difference is, in essence, the elevation difference between the laser hit and tree root elevation. That is, canopy height differences exist without exception because of the uneven ground. The differences will, in turn, influence the extraction of canopy vertices, trunk location, crown, volume and biomass. Extracted forest parameters will be distorted. It is thus imperative to remove the influence of terrain to conduct a more accurate extraction of canopy height. Because the normalized point cloud or CHM is obtained by subtracting the DEM data from DSM data or the raw point cloud, the influence of topography on canopy height varies with individual trees. Therefore, the point cloud of individual trees should be segmented first and foremost. Figure 4 shows a flowchart of the method used. The details are described below.

### 3.2. Processing of LiDAR Data

LiDAR points were classified into ground and non-ground (vegetation) returns with MCC. MCC was designed for classifying LiDAR returns in forested environments occurring in complex terrains [18]. The MCC algorithm operates by discarding returns that exceed a threshold curvature calculated using a surface interpolated [18]. Ranges for initial parameters were selected for MCC based on the scale and curvature ranging from 0.8 to 1.5 and 0.01–0.10, respectively. Ground returns were used to generate DEM intervals of 0.3m. 

Studies have shown that first echo point cloud data can better reflect the forest canopy structure [24]. Therefore, the first echo point cloud was mostly employed in the current paper. Because the first echo point cloud higher than 1.8 m is believed to reflect the actual structure and morphology of the entire crown, these data are usually used for retrieval of forest parameters. Thus echo points of higher than 1.8 m above the ground were generally adopted as vegetation points to avoid the interference of shrub layer [25]. Moreover, point cloud percentiles could reflect the distribution of laser hits [26,27,28]. The proportions of points higher than given thresholds in the total number of points were calculated.

### 3.3. Crown Segmentation

To obtain individual tree height, the initial process is to isolate individual trees and delineate tree crown boundaries. Previous researchers have been done on isolating individual trees using large-scale aerial photos or high-spatial resolution remotely sensed imagery. The methods for isolating individual trees from imagery or photos include: edge detection using scale-space theory [29], local maxima detection [30], local maxima filtering with fixed or variable window sizes [31], local transect analysis [32], and watershed segmentation [33,34]. These methods are mostly based on the assumption that there are “peaks” of reflectance around the treetops and “valleys” along the canopy edges.

Crown segmentation was performed using the eCognition (Definiens Developer 8.7) software package, with multi-resolution segmentation (MS), and a digital orthophoto map (DOM) with a resolution of 0.2 m as input in our study. To avoid over-segmentation as a result of DOM, the segmentation process was applied to a median filtered version of DOM. The median filter applied to the images had a window size of 7 × 7 pixels. The MS algorithm required scale, shape ratio and Compactness ratio as input parameters, a scale parameter associated with the average size of resultant objects, a Shape ratio associated with the shape criterion of homogeneity, and a Compactness ratio associated with the optimization criteria for object shape. The parameters were adjusted as required for each image to account for differences in vegetation structure and distribution. The best segmentation parameters for different forest types were set after repeated experimentation. Segmentation was performed using different parameters: the scale parameter ranging from 10 to 14, the shape ratio ranged from 0.6 to 0.8 and the Compactness ratio ranged from 0.6 to 0.9. The final segments were reviewed manually to ensure quality.

### 3.4. Terrain Correction of Normalized Point Cloud

Point cloud data from individual trees were segmented based on crown boundaries which formed a closed polygon in the segmented DOM. The coordinate planes of centres of gravity were calculated through the weighted height of each point cloud group (Equation (1)). In Equation (1), *x_ig_*, *y_ig_* are the coordinates of centres of gravity in the point cloud group *i*; *A_ij_* are the elevations of points in the point cloud group *i* extracting from DEM; *x_ij_*, *y_ij_*, *z_ij_* are the 3D coordinates of point in the point cloud group *i*; *j* is serial number of grounds *1*, *2*, ..., *n*, while *n* is the total number of points in group *i*:

(1) $$\begin{array}{l} x_{ig}=\frac{\sum_{j=1}^{n}x_{ij}\cdot (z_{ij}-\frac{1}{n}\sum_{j=1}^{n}A_{ij})}{\sum_{j=1}^{n}\left(z_{ij}-\frac{1}{n}\sum_{j=1}^{n}A_{ij}\right)}\\ y_{ig}=\frac{\sum_{j=1}^{n}y_{ij}\cdot (z_{ij}-\frac{1}{n}\sum_{j=1}^{n}A_{ij})}{\sum_{j=1}^{n}\left(z_{ij}-\frac{1}{n}\sum_{j=1}^{n}A_{ij}\right)} \end{array}$$

For individual trees and forest gaps, the calculated coordinates represent the peak of the tree’s canopy relative to the ground and the geometrical centre of the gap, respectively. When a tree stands upright, its peak is directly over the base of the tree. Because this paper focuses on the effects of terrain to canopy height and forest biomass, the forest gap point cloud was removed by setting a height threshold. Finally, elevation values of centres of gravity *z_ig_*, namely the elevation values of the tree base, were extracted from DEM according to their same plane coordinates. Additionally, the difference between *z_ig_* and elevation of the point cloud in each group was considered to be the normalized point cloud after correction.

### 3.5. Individual Tree Locations Extracted

Before correction, CHM I was generated by subtracting DEM data from DSM data or the raw point cloud data. Normalized point cloud data based on individual tree crown segmentation was used to generate CHM II after correction through the Inverse Distance Weighted (IDW) interpolation method [35]. Individual tree crowns and individual tree locations were extracted to assess deviation caused by terrain of CHM retrieval from LiDAR data before and after correction. Individual tree crowns were also extracted by the canopy morphological-controlled watershed method from both CHMs before and after correction [36]. Morphological crown control was introduced to ensure that the watershed results are accurately located in the crown area. Additionally, both CHMs were used to extract individual tree locations through the region growing method [36]. The local maxima algorithm is used to identify potential tree positions in crown area.

Regression analysis was conducted using Microsoft Excel to assess the correlation between the slope gradient and the proportions of points with different thresholds of canopy height differences before and after correction.

### 3.6. Assessing the Consequence of Correcting Topographic Effects

We apply a mean stand height weighting scheme, named the Lorey’s height (basal-area-weighted average height) [37], which already is in common use in forestry. Lorey’s height is defined by Equation (2):

(2) $$Lh=\frac{\sum G_{i}\cdot h_{i}}{\sum G_{i}}$$

where *Lh* is the Lorey’s height (basal-area-weighted average height), *G*_i_ is the basal area of stem i, and *h*_i_ is the height of stem i.

Stepwise multiple regressions were used to find a relationship between canopy heights variables and field surveyed Lorey’s height. Canopy heights variables from the LiDAR data before and after terrain correction were used as independent variables in the regression analysis. Two models were built respectively (Equation (3)). We used a K-fold cross-validation procedure (JMP 10, SAS Institute, Cary, NC, USA) to identify the most appropriate dimension for the regression models. This procedure splits the dataset into K groups and fits a regression model to all groups except one. The model giving the best validation statistic is chosen as the final model. This method is best for small data sets, because it makes efficient use of limited amounts of data:

(3) $$Lh=\beta_{0}+\beta_{1}h_{10}+\beta_{2}h_{20}+\cdots +\beta_{9}h_{90}+\beta_{10}h_{mean}+\varepsilon$$

where *h_10_*, *h_20_*, …, *h_90_* and *h_mean_* is 10%, 20%, …, 90% height quantile and average height of airborne LiDAR point cloud respectively; *β_0_*, *β_1_*, *β_2_*, …, *β_9_* and *β_10_* is the coefficient of model respectively; *ε* is the error of model.

Stepwise variable selection and the maximum K-fold R-square improvement variable selection techniques were applied to select the LiDAR-derived variables to be included in the models [24]. The two Lorey’s height estimation models based on 41 plots were named Models I and II, respectively. In assessing deviation caused by terrain of canopy height, we report *R*^2^ for statistically significant (at *p* < 0.001) regressions, the *RMSE*, equation intercept and coefficients, and the maximum K-fold R-square.

## 4. Results

### 4.1. CHMs before and after Correction

**Figure 5:**
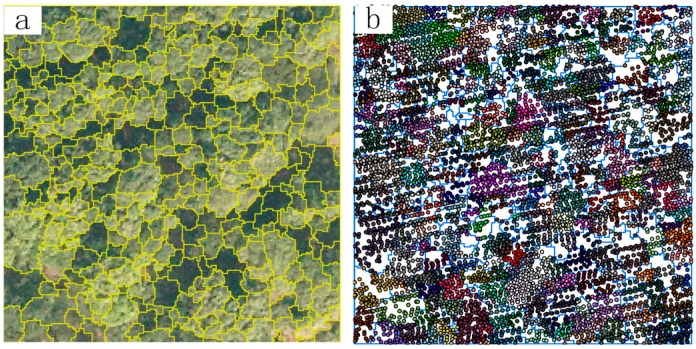
Multi-resolution segmentation in Plot No. 34: (**a**) segmented digital orthophoto map; (**b**) segmented point cloud.

Figure 5 shows the results of crown and point cloud analysis segmented by MS in plot No. 34. The MS segmentation parameters were set to the Scale parameter of 14, the Shape ratio is 0.6 and the Compactness is 0.8. The point cloud was separated into both individual tree and forest gap point clouds (Figure 5b).

**Figure 6:**
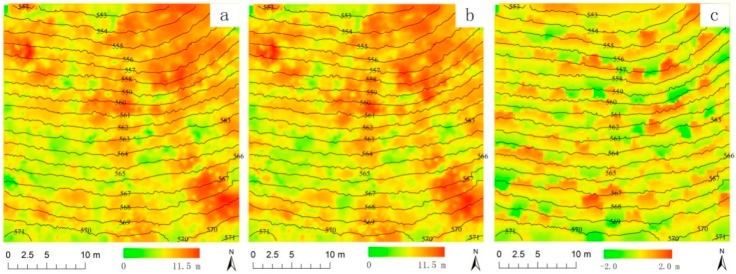
Canopy model heights (CHMs) before and after correction and difference image in plot No. 34: (**a**) CHM I before correction (**b**) CHM II after correction; (**c**) image of the differences.

Figure 6a,b shows CHM I and II based on the IDW interpolation before and after correction. The differences between CHMs before and after correction ranged from −2 m to 2 m (Figure 6c). The positive and negative values appear alternately along the direction of slope. The differences are negative at the upslope side from the centre point of the trunk, and are positive at the downslope side.

### 4.2. Impact of Topography on Individual Tree Extraction

**Figure 7:**
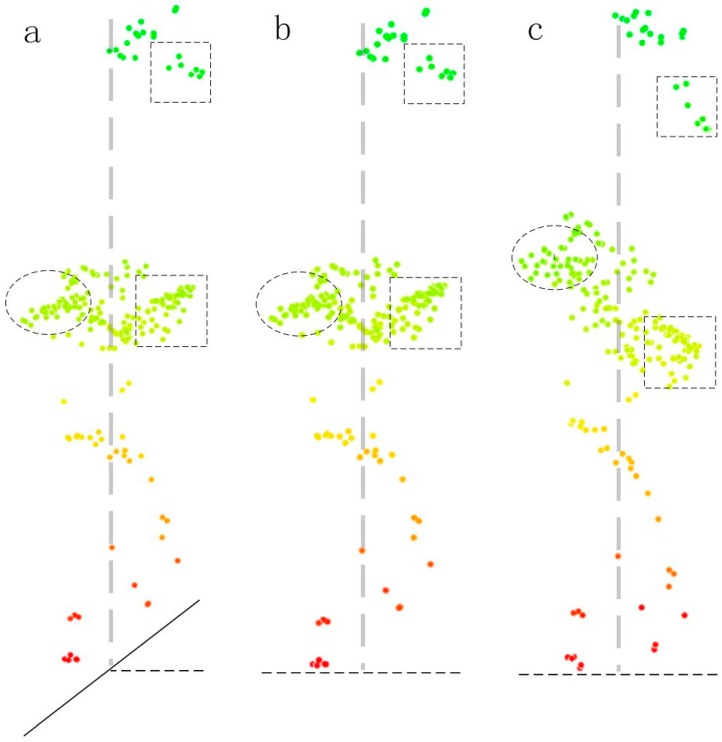
Point cloud of individual tree canopies: (**a**) raw point cloud; (**b**) new normalized point cloud; (**c**) normalized point cloud by a conventional method.

Figure 7 shows the point cloud of an individual tree in the study area. The ground slope gradient is 38.2°, tree height is 24.4 m, and crown radius is 6.5 m and 5.5 m in cross direction. Figure 7a shows the raw point cloud data; Figure 7b presents the new normalized point cloud proposed in this paper; Figure 7c shows normalized point cloud data by the conventional method, *i.e.*, by subtracting the DEM from the raw point cloud. The long dashed lines represent the trunk. Point cloud data in Figure 7a,b are highly consistent with tree morphology, while Figure 7a,c are quite different. To be specific, the point cloud data in the oval box on the left side of the dashed line of Figure 7c are higher than the raw point cloud, whereas those in the square box on the right side of the dashed line are lower than the raw point cloud. The left and right sides of the dashed line is the downslope and upslope sides, respectively. In other words, the conventional normalized point cloud, which is the differences between raw point cloud and DEM, actually increases the modelled downslope side of the canopy by up to 2.23 m; while the upslope side of the canopy is lowered by up to −2.34 m. The extent to which the canopy height is raised or lowered is related to the slope gradient and the horizontal distance between the point cloud and the trunk. Greater gradients and longer distances from the point cloud to the trunk cause greater height differences. Points close to the trunk centre display nearly zero difference.

**Figure 8:**
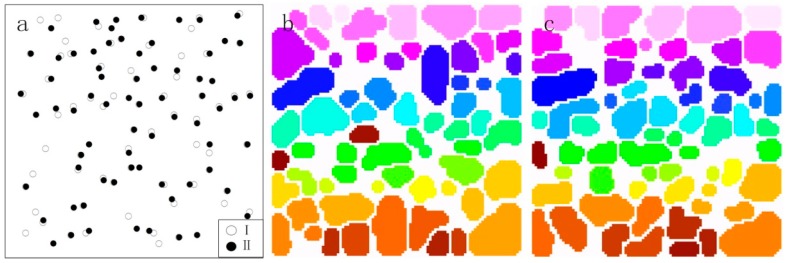
Individual tree location and crown comparison: CHM I *vs.* CHM II. (**a**) Individual tree locations extracted from CHM I and CHM II; (**b**) crowns extracted from individual trees based on CHM I; (**c**) crowns extracted from individual trees based on CHM II.

CHM is a key factor affecting the accuracy of parameter extraction from individual trees in that CHM directly influences the extracted location and crown of single trees. CHM is acquired from the normalized point cloud after interpolation. Figure 8 shows individual tree crowns and individual tree locations extracted. The results showed differences in both the number of trees and tree positions: 62 trees from CHM I and 63 from CHM II and a maximum distance difference of 1.57 m in terms of tree location (Figure 8a). The number of crowns based on CHM I and CHM II were 71 and 68, respectively, representing some integrations and separations. Apart from different crown numbers, there was also a considerable amount of difference in the shape and size of crowns (Figure 8b,c).

Because the traditional normalization method results in raising or lowering a point cloud, the normalized point cloud shows a much different morphology from the raw point cloud data, making it impossible to capture the accurate canopy structure of trees. There would be significant variation in the canopy vertices, individual tree locations and crowns extracted using these two methods.

### 4.3. Impact of Topography on Plot-Level Canopy Height

Point clouds in plot-level data areas are often used to establish biomass estimation models. Normalized point clouds and related statistics at the plot level have important effects on the precision of forest biomass models making it important to analyse how topography impacts canopy height at the plot level so that the precision of biomass estimation can be improved.

**Figure 9:**
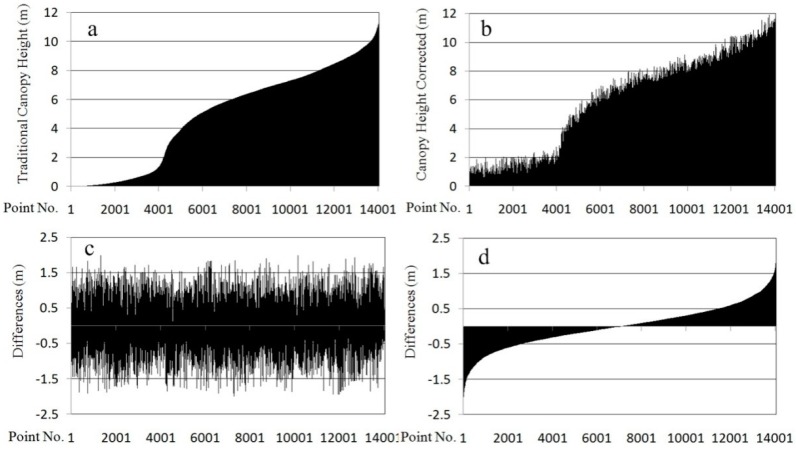
Canopy height before and after correction and canopy height differences in plot No. 34: (**a**) canopy height before correction; (**b**) canopy height after correction; (**c**) canopy height differences before and after correction; (**d**) canopy height differences in ascending order.

Figure 9a–c shows canopy heights in plot No. 34 before and after correction in ascending order as well as their differences. Figure 9d presents canopy height differences in ascending order; canopy height differences before and after correction, *i.e.*, terrain-triggered canopy height differences, are within ±2.0 m, and show a symmetrical distribution (Figure 9). This is the main reason that the ground slopes are basically uniform, and canopy has a symmetrical form with the trunk as the axis. The normalized point cloud at the downslope side was elevated with the normalized point cloud by the conventional method, so the differences are positive. Conversely, the normalized point cloud at the upslope side was lowered in elevation, so their differences are negative. In Figure 9b, with a canopy height showing the new normalized point cloud data of within ±1.8 m that corresponds to shrub or ground points, data within ±1.8 m is excluded by setting a threshold when forest parameters are extracted, such as tree height, crown, height to the first live branch, canopy density, biomass and so on. In other words, canopy height of less than ±1.8 m has nothing to do with forest parameters. The remaining point cloud whose canopy height is taller than 1.8 m after correction reflects the natural form of the canopy structure, and is thus conducive to subsequent extraction of parameters as listed above.

The absolute value of terrain-caused canopy height differences of all plots was calculated and analysed. A difference threshold, *k*, was set at 0.3, 0.5, 0.8, 1.0, 1.2, and 1.5 m for various tests. Average values of differences above each threshold *k* are labelled as mean_0.3_, mean_0.5_, mean_0.8_, mean_1.0_, mean_1.2_, and mean_1.5_, respectively. The proportions of the point counts of canopy height differences higher than a given threshold *k* in the total number of points are labelled as p_0.3_, p_0.5_, p_0.8_, p_1.0_, p_1.2_, and p_1.5_, respectively. The plots were numbered based on their slope gradients in ascending order.

**Figure 10:**
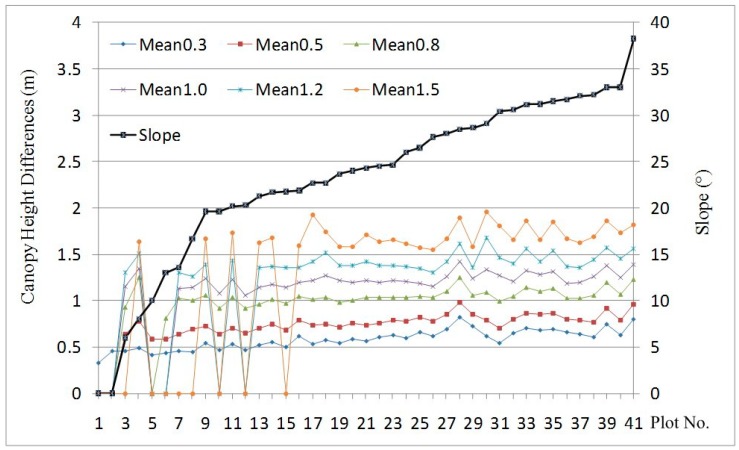
Average values of terrain-triggered canopy height differences *versus* slope.

Figure 10 shows average differences before and after canopy height correction. A zero average difference indicates no difference is greater than the corresponding threshold *k*. Based on the statistical results, the average values of mean_0.3_, mean_0.5_, mean_0.8_, mean_1.0_, mean_1.2_, and mean_1.5_ of all plots vary: 0.58, 0.72, 0.97, 1.11, 1.21, and 1.28 m, respectively, and the maximum values of all plots were 0.83, 0.98, 1.25, 1.43, 1.67, and 1.95 m, respectively. In general, the average value of differences increases as the slope becomes steeper (Figure 10). However, when the slope is ≥22°, the average canopy height difference barely increases. This can be mostly explained by the small crown radius within the plots which is average 3.5 m. Smaller crowns indicate that slope has less impact on the canopy height than larger crowns. Additionally, the canopy height differences tend to be symmetrical, which means that similar characteristics are observed regardless of whether the differences are negative or positive.

**Figure 11:**
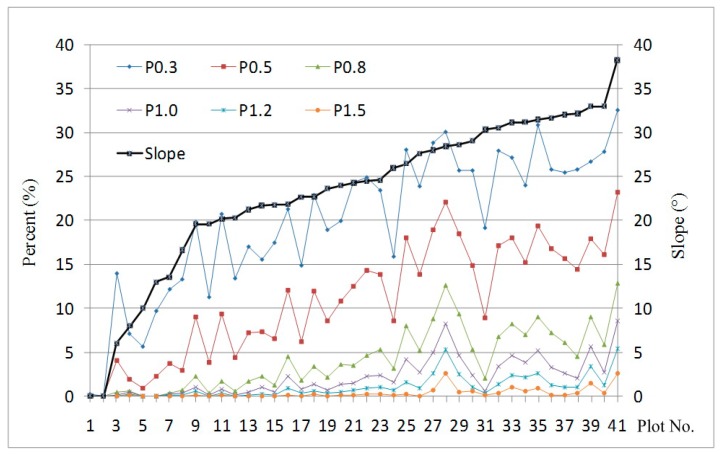
Proportions of points with differences greater than given thresholds *versus* slope.

Figure 11 shows the proportions of points higher than given thresholds for the total number of points. Average values of p_0.3_, p_0.5_, p_0.8_, p_1.0_, p_1.2_, and p_1.5_ of all plots were 20.0%, 11.0%, 4.2%, 2.2%, 1.1%, and 0.35%, respectively, and maximum values of p_0.3_, p_0.5_, p_0.8_, p_1.0_, p_1.2_, and p_1.5_ of all plots were 32.6%, 23.2%, 12.9%, 8.5%, 5.3%, and 2.6%, respectively. When the slope increases from zero to 38.2°, the proportions of points with differences larger than 0.3, 0.5, 0.8, 1.0, 1.2 and 1.5 m increase from 0% to 32.6%, 23.2%, 12.9%, 8.5%, 5.3% and 2.6%, respectively (Figure 11). Overall, the proportion increases as the slope increases, but it decreases as the threshold grows.

**Figure 12:**
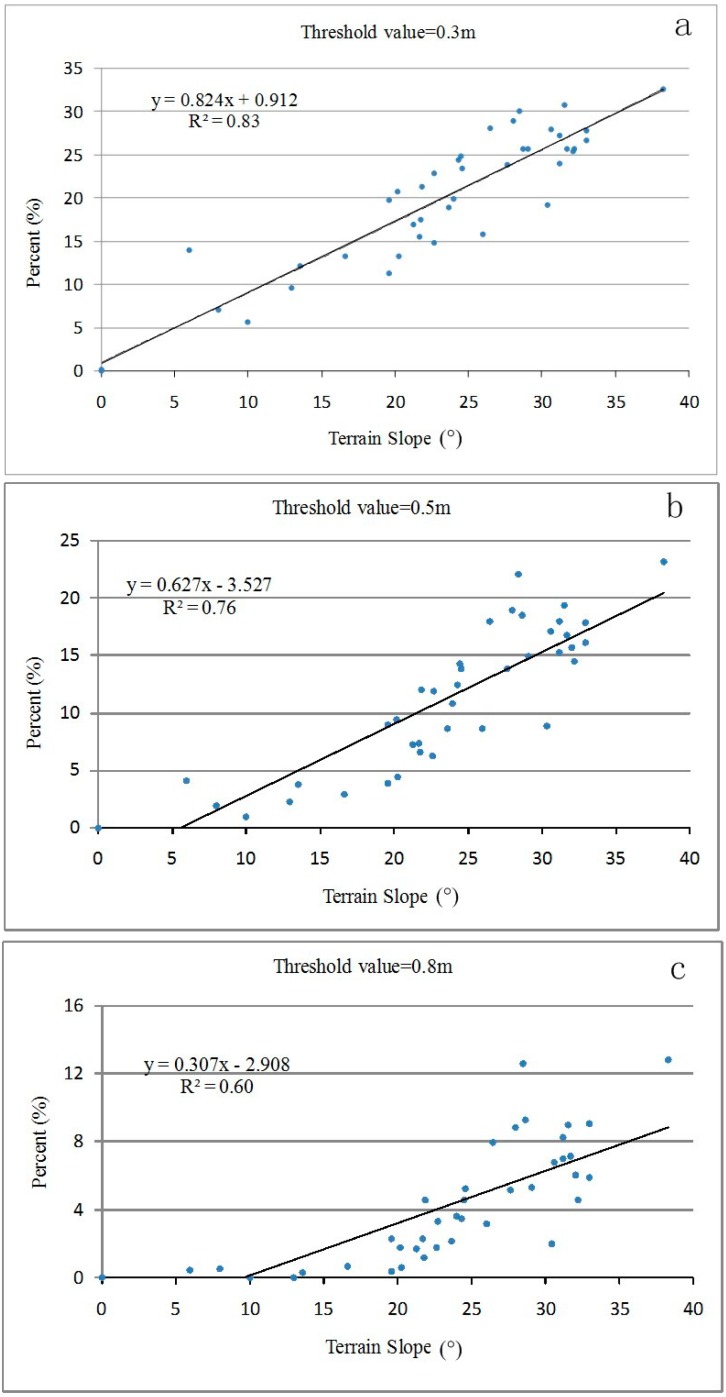
Regression relationships between slope and proportions of points with differences greater than selected thresholds: (**a**) threshold = 0.3 m; (**b**) threshold = 0.5 m; (**c**) threshold = 0.8 m.

Meanwhile, regression analysis shows a strong correlation between the slope gradient and the proportions of points with differences greater than 0.3, 0.5 and 0.8 m (Figure 12); the coefficient of determination *R^2^* is 0.83, 0.76, and 0.60 (n = 41), respectively. But when the threshold is increased to ≥1.0 m, the correlation weakens. This is also attributable to smaller crowns, which means terrain creates less impact. Larger crowns apparently lead to more slope-caused canopy height differences.

### 4.4. Consequence of Correcting Topographic Effects

**Figure 13:**
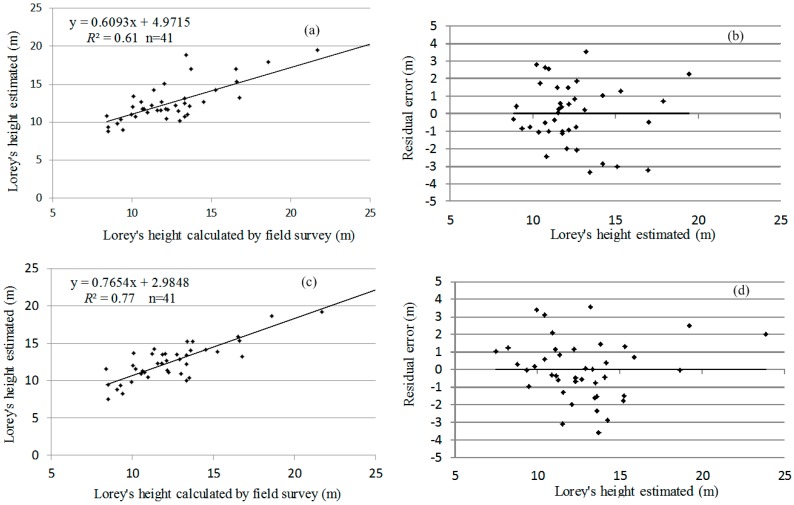
Correlation and residual error of the estimated Lorey’s height before and after terrain correction. (**a**) Correlation of the estimated Lorey’s height before terrain correction; (**b**) Residual error of the estimated Lorey’s height before terrain correction; (**c**) Correlation of the estimated Lorey’s height after terrain correction; (**d**) Residual error of the estimated Lorey’s height after terrain correction.

To assess the consequence of correcting topographic effects on forest canopy height, the two best regression models were chosen and built according to the maximum R-square stopping rule from 10-fold cross validation, namely Model I and Model II (Table 3). The height of the canopy at the 10th, 40th, 60th, 90th percentile and average height were selected in Model I, the determination coefficient *R*^2^ is 0.61, corresponding adjusted *R*^2^ is 0.58, *RMSE* is 2.24 m, the response mean is 12.72 m, and the maximum K-fold *R*^2^ is 0.52. The height of the canopy at the 10th, 40th, 50th, 70th, 80th, 90th percentile and average height were selected in Model II, the determination coefficient *R*^2^ is 0.77, corresponding adjusted *R*^2^ is 0.71, *RMSE* is 1.86 m, and the response mean is 12.72 m, and the maximum K-fold *R*^2^ is 0.62 respectively (Figure 13). Experimental results show that the correlation between Lorey’s height calculated by filed survey and canopy height quantiles after terrain correcting is better than before terrain correcting, which reveals that normalized canopy height point cloud after the terrain correction is closer to the natural formation of forest canopy. It can demonstrate that the method of terrain correction restores natural forms of forest canopy.

**Table 3:** Regression coefficients, estimated values and precision indexes of the models.

Coefficients	Model I ( n = 41)				Model II ( n = 41)			
	Estimated Values	Error Sum of Squares (SS)	F Ratio	P > F	Estimated Values	Error Sum of Squares (SS)	F Ratio	P > F
*β_0_*	1.382009	0.0	0.000	1	0.814054	0.0	0.000	1
*β_1_*	−3.0262	36.37898	8.80696	0.005382	−3.33578	34.44685	9.895305	0.003499
*β_2_*								
*β_3_*								
*β_4_*	2.62023n7	34.95306	8.46176	0.006263	3.454034	26.42372	7.590555	0.009475
*β_5_*					−6.26901	21.0267	6.04019	0.019406
*β_6_*	−3.33947	40.49818	9.804176	0.003506				
*β_7_*					8.454889	19.45856	5.589724	0.024103
*β_8_*					−7.81642	29.14998	8.37371	0.006697
*β_9_*	0.569135	23.73004	5.744788	0.022012	1.141369	52.70873	15.14126	0.000458
*β_10_*	3.614737	19.06262	4.614855	0.038697	5.001009	25.61142	7.357213	0.010527

## 5. Discussion

This paper offers a correction method of terrain-induced canopy height differences under the premise that the trees stand vertically. Of course, the suggested correction may not be applicable to tilting trees. Accurate measurement of the canopy height of tilting trees is more complicated, although this can be performed using auxiliary ground measurements [7].

Another premise of this paper is that the point clouds representing individual trees must be generated by DOM segmentation. The segmentation process relies on user-specified parameters regarding the scale, shape ratio and Compactness ratio in this study. When segmenting crown from remote sensing image and aerial photography image with high resolution, these methods are mostly based on spectral properties. However, the “peaks” and “valleys” are not always distinct since canopy reflectance is affected by various factors such as illumination conditions, canopy spectral properties, and complex canopy structure.

Meanwhile, there may be overlapping point clouds in complex terrain and/or closed-canopy forest. Moreover, because the airborne LiDAR scanning was done at a large side-viewing angle, shadows fall into the DOM image. Therefore, only point clouds within the plots were segmented in this study and there may be some errors in the segmentation. Nevertheless, the DOM segmentation was done after repeated experimentation, and we believe that point clouds of single trees obtained this way had only minor errors. Segmentation of point clouds for the entire region is not currently feasible and warrants further study.

The crown segmentation is the key procedure for terrain correction of forest canopy height retrieval using airborne LiDAR. Another crown segmentation method bases on airborne LiDAR data. Compared with passive imaging, LiDAR has the ability of directly measuring the three-dimensional coordinates of canopies. Therefore, the geometric, rather than spectral, “peaks” and “valleys” can be detected. Researchers have applied LiDAR data into crown segmentation and individual tree isolation directly [38,39,40]. The crown segmentation methods using airborne LiDAR can be divided into two kinds, and they are based on discrete point cloud and with surfaces derived from point cloud. The first method segments crown using the point cloud directly. The later method usually transforms the point cloud into a raster. The first and final returns were used for generating the DSM and the CHM. The CHM has inherent errors and uncertainties from a number of sources. Such LiDAR-derived surface models often contain so-called “pits” which occur. The pit-free algorithm can be used for generating the pit-free CHM [41]. Meanwhile, spatial error can be introduced during the interpolation process from the point cloud to the gridded height model [7]. The uncertainty introduced by the interpolation method can affect the accuracies of the segmentations. Compared with using CHM, the crown segmentation methods using discrete point cloud can take advantage of the full 3D structure inherent in the LiDAR point cloud. Many approaches using point clouds have been developed to segment crown, such as region growing [42], marker-controlled watershed segmentation [43], combination of pouring algorithm and knowledge-based assumptions [44], adaptive clustering [45]. Jakubowski *et al.* [46] compared a 3D lidar point cloud segmentation algorithm to an object-based image analysis (OBIA) of CHM to determine the difference between the two types of approaches. The two approaches delineated tree boundaries that differed in shape: the lidar-approach produced fewer, more complex, and larger polygons that more closely resembled real forest structure. Effectively, the lidar segmentation method tended to under-segment and under-detect trees, while the OBIA method over-segmented the trees. However, there are over-segmentation and under-segmentation in segmenting forest crown using remote sensing image and using airborne LIDAR data. Further research is necessary to improve precision of segmentation and to automate the segmentation process. However, the correction method based on individual tree crown segmentation is only applicable on the plot scale, because of overlapping point clouds caused by complex terrain, closed forest canopies, and other reasons. Consequently, the segmentation of point clouds representing the entire region is worth further study.

Geoscience Laser Altimeter System (GLAS, footprint size = ~70 m), with a larger-footprint and wide spatial coverage, has provided practical means for monitoring various global forest attributes. The sloped terrain generally lengthens the full extent of GLAS waveform and decreases the level of laser energy at the forest canopy and ground peaks [47]. Lee *et al.* [48] found that the larger footprint size and greater slope tend to generate more errors in the retrieved lidar forest canopy heights. Park *et al.* [49] found that both Laser Vegetation Imaging Sensor (LVIS, footprint size = ~20 m) and GLAS campaigns could be benefited from the physical correction approach, and the magnitude of accuracy improvement was determined by footprint size and terrain slope, off-nadir pointing angle. Our results indicate that airborne LiDAR data also can be benefited from topographic correction. It can be concluded that both large and small footprint LiDAR data will encounter high terrain slop problem, and topographic correction is required.

## 6. Conclusions

Canopy height serves as basic data for the extraction of forest parameters using LiDAR. However, canopy height obtained by subtracting DEM data from DSM data contains errors because of the influence of terrain. This paper proposes a method for correcting terrain-derived canopy height differences based on individual tree crown segmentation. The new normalized point clouds are very consistent with raw point clouds morphologically. The method can obtain accurate normalized canopy heights which recover the natural structure of the canopy.

The results show canopy height differences are connected with slope gradients and crown radius. Steeper slopes and larger crowns cause greater differences. For individual trees, terrain influences the estimated canopy height of individual trees when the DEM is subtracted from the DSM, the downslope side of the tree trunk is elevated and the upslope side is lowered. For plot scale measurement, a strong correlation exists between the slope gradient and the proportions of points with differences higher than 0.3, 0.5 and 0.8 m, with the coefficient of determination *R*^2^ at 0.83, 0.76, and 0.60 (n = 41), respectively.
